# Understanding the uptake of a clinical innovation for osteoarthritis in primary care: a qualitative study of knowledge mobilisation using the i-PARIHS framework

**DOI:** 10.1186/s13012-020-01055-2

**Published:** 2020-10-28

**Authors:** Laura Swaithes, Krysia Dziedzic, Andrew Finney, Elizabeth Cottrell, Clare Jinks, Christian Mallen, Graeme Currie, Zoe Paskins

**Affiliations:** 1 Impact Accelerator Unit, Versus Arthritis Primary Care Centre, School of Medicine, Keele University, Staffordshire, ST5 5BG UK; 2 Versus Arthritis Primary Care Centre, School of Medicine, Keele University, Staffordshire, ST5 5BG UK; 3 Entrepreneurship & Innovation, Organising Healthcare Research Network, Warwick Business School, The University of Warwick, Coventry, CV4 7AL UK

**Keywords:** Knowledge mobilisation, Implementation; Primary care, Osteoarthritis, Qualitative, i-PARIHS, Theoretically informed

## Abstract

**Background:**

Osteoarthritis is a leading cause of pain and disability worldwide. Despite research supporting best practice, evidence-based guidelines are often not followed. Little is known about the implementation of non-surgical models of care in routine primary care practice. From a knowledge mobilisation perspective, the aim of this study was to understand the uptake of a clinical innovation for osteoarthritis and explore the journey from a clinical trial to implementation.

**Methods:**

This study used two methods: secondary analysis of focus groups undertaken with general practice staff from the Managing OSteoArthritis in ConsultationS research trial, which investigated the effectiveness of an enhanced osteoarthritis consultation, and interviews with stakeholders from an implementation project which started post-trial following demand from general practices. Data from three focus groups with 21 multi-disciplinary clinical professionals (5–8 participants per group), and 13 interviews with clinical and non-clinical stakeholders, were thematically analysed utilising the Integrated Promoting Action on Research Implementation in Health Services (i-PARIHS) framework, in a theoretically informative approach. Public contributors were involved in topic guide design and interpretation of results.

**Results:**

In operationalising implementation of an innovation for osteoarthritis following a trial, the importance of a whole practice approach, including the opportunity for reflection and planning, were identified. The end of a clinical trial provided opportune timing for facilitating implementation planning. In the context of osteoarthritis in primary care, facilitation by an inter-disciplinary knowledge brokering service, nested within an academic institution, was instrumental in supporting ongoing implementation by providing facilitation, infrastructure and resource to support the workload burden. ‘Instinctive facilitation’ may involve individuals who do not adopt formal brokering roles or fully recognise their role in mobilising knowledge for implementation. Public contributors and lay communities were not only recipients of healthcare innovations but also potential powerful facilitators of implementation.

**Conclusion:**

This theoretically informed knowledge mobilisation study into the uptake of a clinical innovation for osteoarthritis in primary care has enabled further characterisation of the facilitation and recipient constructs of i-PARIHS by describing optimum timing for facilitation and roles and characteristics of facilitators.

**Supplementary Information:**

The online version contains supplementary material available at 10.1186/s13012-020-01055-2.

Contributions to the literature

- This study makes a theoretical contribution to i-PARIHS by illuminating different roles and activities of facilitation in primary care and illustrating how public contributors can be recipients and facilitators of implementation
- ‘Instinctive’ facilitation occurred when key individuals, including patients, were unaware of their role in knowledge mobilisation, utilising tacit knowledge to support implementation
- Conditions deemed ‘low-priority’ by stakeholders may require more dedicated facilitation
- The end of a trial is an important opportunity for facilitation to trigger knowledge mobilisation and catalyse implementation
- An inter-disciplinary knowledge-brokering service within a clinical-academic unit provided infrastructure and resource to facilitate knowledge mobilisation and support implementation

## Background

Osteoarthritis (OA) is the most common joint disorder in the Western world. It is a leading cause of pain, loss of function and disability worldwide and is predominantly managed in primary care [1]. Despite international evidence-based guidelines that support best practice, management of OA remains suboptimal [2, 3]. Core approaches for managing OA, such as exercise, are underutilised and the quality of care for adults with OA is inconsistent [4].

Internationally, effective non-surgical models of OA care do not inevitably translate to improved clinical practice that benefits patients [5–7]. Where post-trial implementation does occur, little is known about how this is achieved in different contexts [8].

Factors that influence implementation of models of care, across a range of conditions in primary care, have been identified [9, 10]. Public awareness, resources, philosophy of care and ease of implementation are possible barriers and facilitators of implementation [9]. Strategies such as educational meetings, visits and audit have the potential to optimise the process [10]. These factors operate at the level of systems, organisations, professionals and innovations; however, in the case of OA, the congruence of the innovation with healthcare professionals’ (HCPs) attitudes and perceived role appears to be important [11].

Implementation of empirically tested approaches can be challenging if wider influences are not accounted for. Failed implementation efforts pose health, economic and opportunity costs [12]. Knowledge mobilisation (KM) is a perspective that recognises the non-linearity associated with the dynamic nature of creating, sharing and using knowledge across practice domains to improve outcomes and efficiency for relevant stakeholders [13, 14]. The complexity of KM is compounded by the interaction of multiple systems, policy, organisational and personal factors [15]. A recent systematic review of the factors that influence implementation of evidence-based guidelines for OA in primary care identified a paucity of studies and illustrated the challenges in identifying and mobilising knowledge relevant to policy, practices and individuals to optimise implementation [8].

This study aimed to understand the uptake of a clinical innovation for OA and explore the transition of knowledge from a clinical trial to implementation from a KM perspective, using the Integrated Promoting Action on Research Implementation in Health Services (i-PARIHS) framework in a theoretically informative approach.

## Methods

### Overview of context and innovation

The Managing OSteoArthritis in ConsultationS (MOSAICS) trial was a cluster randomised controlled trial, to investigate the effectiveness of a model OA consultation in improving the uptake of core recommendations, described by the National Institute for Health and Care Excellence (NICE) OA guidelines (NICE, 2008, updated 2014), in UK primary care [16] (Table 1).

**Table 1:** MOSAICS study context

Context to the Managing Osteoarthritis in Consultations (MOSAICS) Study	
Overview	MOSAICS was an investigation of the feasibility, acceptability and impact of implementing the National Institute for Health and Care Excellence (NICE) osteoarthritis (OA) Guideline [16]. MOSAICS was informed by the NICE guidelines (NICE 2008, updated 2014) [17] and the intervention development was guided by theory and shaped by clinical and academic stakeholders and public contributors [18, 19]. The aim of MOSAICS was to evaluate the clinical and cost-effectiveness of a ‘model OA consultation’—a complex intervention designed to increase adherence to national guidelines for OA management in primary care. Theoretical approaches used to inform MOSAICS: •Implementation of Change model [20] to guide the overall approach. The model comprises five steps: developing a concrete proposal for change; undertaking an analysis of current practice; developing and selecting ways to change practice and undertaking and evaluating the implementation plan •The Theoretical Domains Framework [21] consists of 12 domains and was used to identify relevant domains of behaviour change and to understand which factors would impede or facilitate the intended healthcare professional behaviour change. •The Whole Systems Informing Self-management Engagement (WISE) approach underpinned the development of the MOSAICS model [22]: relevant and accessible patient information, professionals responsive to the needs of patients and good access to care services.
Context	A mixed methods research study incorporating a population survey, cluster randomised controlled trial, consultation, and medical record review, and an evaluation of a model OA consultation intervention and training, conducted in general practice primary care in England
Innovation	Components of the trial intervention: i) An OA Guidebook written by patients and health professionals for patients to provide patient-centred and evidence-based information ii) A model OA consultation for primary care to deliver NICE interventions for people aged 45 years or older presenting to the practice with peripheral joint pain iii) Training for GPs and practice nurses to deliver the model consultation iv) The development and capture of quality indicators of care (through an OA e-template and self-reported questionnaire) The MOSAICS model consisted of three components: (i) an initial consultation with a GP, followed by (ii) up to four consultations with a practice nurse in an OA clinic, with (iii) the Keele OA Guidebook to support care. The evidence-based intervention was designed to provide relevant written information for patients, along with support in undertaking muscle strengthening exercises, increase physical activity and weight loss (if appropriate).
Recipients	GPs and practice nurses in general practices involved in the trial
Facilitation	Components of the training package delivered in the MOSAICS trial [18, 19]: **Theoretical approach:** Training package development informed by the Theoretical Domains Framework **Content:** Provided information on establishing the current practice, core NICE recommendations for OA (diagnosis, written information [the OA guidebook], exercise and physical activity, healthy eating, pain management), history taking and self-management support **Delivery:** Training incorporating a mixture of didactic and interactive sessions (including the use of simulated patients) which were learner centred and facilitated by local opinion leaders. **Duration of training for intervention practices**: GP training - four sessions (2 h ×3, 1 h ×1). Practice nurse training – 4 days **Duration of training for control practices:** Three sessions over a 3-week period, after completion of trial. The first two sessions of GP training comprised 2× lunchtime sessions and for Practice nurses, 2× 1-day workshops. The final session for all staff was a focus group discussion with the whole practice, led by a facilitator who had been involved in MOSAICS as a rheumatology advisor.

The MOSAICS innovation consisted of four components:

1. A model OA consultation for primary care to deliver NICE recommendations (comprising a general practitioner (GP) consultation to make, give and explain the diagnosis of OA and up to four consultations with a practice nurse (PN) to support self-management [18]
2. An OA guidebook providing high-quality written information designed by patients [23]
3. GP and nurse training to deliver the model consultation [24, 25]
4. OA e-template to record OA-associated codes in electronic health records [26]

To recognise and reward their participation in the study, the four control practices in the MOSAICS trial received whole-practice training on the key components of the enhanced OA consultation, at trial end, prior to the results being known. The training was a condensed version of that given to the intervention practices but incorporated practice-based learning that had arisen during the trial [27, 28]. A facilitated focus group discussion took place at the end of the control practice training to gain feedback and explore its potential for changing practice.

Subsequently, one of the control practices continued to implement the innovations to improve the consistency and quality of OA care within their practice. This primary care-led demand to implement the MOSAICS innovations resulted in the launch of the Joint Implementation of Guidelines for Osteoarthritis in the West Midlands (JIGSAW) project (Table 2). In JIGSAW, the innovation remained largely similar to MOSAICS, but additional strategies to support implementation across a wider UK context were developed and offered, informed by practice-based learning from MOSAICS. An Impact Accelerator Unit (IAU) evolved within the academic institution that conducted MOSAICS, to support these activities. Support strategies for practices to operationalise JIGSAW included a central point of contact for queries and problems, inter-disciplinary champions, workshops and a modified training package.

**Table 2:** JIGSAW implementation project context

Context to the Joint Implementation of GuidelineS for osteoarthritis in the West midlands (JIGSAW) implementation project	
Overview	In 2013, JIGSAW identified 15 general practices in the local Clinical Commissioning Group (CCG) in England to be pilot sites, with the initial aims of: 1. Testing out the practicalities of implementing the model osteoarthritis (OA) consultation developed in the MOSAICS study 2. Improving the alignment of OA care with current recommendations through the provision of innovations (OA e-template, training, enhanced consultation and patient materials) 3. Supporting primary care with the systematic implementation of international guidelines and quality standards for OA at a practice level 4. Reducing clinical variation, and improving evidence-based practice, patient satisfaction and clinical outcomes Theoretical approach used to inform JIGSAW: •Normalisation Process Theory (NPT) – NPT can be used to describe, assess and enhance implementation activity by explaining the processes in which complex interventions become sustained or routinely embedded, in their social context (healthcare practice) [29]. NPT was used in the MOSAICS study qualitative evaluation as a framework for exploring aspects of adoption and implementation of the innovation [27] and as a result informed JIGSAW.
Context	The JIGSAW implementation project was initiated as a result of a primary care led demand to implement the MOSAICS innovation. Professionals within practices in the MOSAICS trial who had delivered the enhanced OA consultation recognised the benefits of the approach in improving the quality of care for people with OA and that the innovations had a positive impact for example with regards to clinicians knowledge and confidence in managing the condition and increased uptake of some quality standards of OA care [30]. Practice-based learning enabled adaptation of the innovation (training component) that was required to scale-up empirically tested MOSAICS innovations (from a research trial) into ‘real world’ primary care (JIGSAW).
Innovation	The JIGSAW approach required practices to implement the four key innovations that were delivered in MOSAICS: clinician training, structured consultations with follow-up, patient information in the form of the OA guidebook and the e-template. These innovations can be delivered flexibly in a way that suits local healthcare context. The refined JIGSAW training package comprised a one-hour all practice meeting and a 2-day primary care nurse training programme. The training content included; what is OA, how should OA be explained, core management of OA and goal setting with patients. Much of the training was interactive including a session with simulated patients.
Recipients	GPs and practice nurses
Facilitation	Facilitation was led by an inter-disciplinary team who provided a knowledge brokering service nested within a clinical academic unit of expertise. The team comprised: •Academic leadership – recognised international leaders in OA research, particularly developing models of care for the management of OA •Specific management expertise including project management and health services management •Patient and public involvement and engagement (PPIE) supported by a knowledge broker •Clinical leadership and expertise – clinical champions with local and national profile •Education expertise •Information technology expertise Services offered and support provided (knowledge mobilisation methods and facilitation activities) by the team included: •Stakeholder engagement •Applying for and securing funding to offer free training to local practices •Hosted workshops and events for professionals and the public based on the research training for the MOSAICS trial to share practice-based learning •Profession specific and lay JIGSAW champions •Conducted whole practice meetings with relevant champions •Supported Clinical Commissioning Group (CCG) led implementation as part of a Locally Enhanced Service (LES) in one area •PPIE liaison in general practices

### Theoretical underpinning

Implementation theories provide an important conduit between empirical observations and both theoretical and empirical knowledge; they help to understand the research-primary care practice interface ensuring that all key influences are considered [9, 31]. A theoretically informative approach is advocated to develop, refine and advance conceptual knowledge [31], whereby research findings are used to develop new theoretical insights rather than simply using theory to explain findings.

Pre-data collection, we reviewed applicable theories and conducted a stakeholder workshop to discuss the ‘fit’ of KM theories and frameworks with KM practice. Following these activities, the i-PARIHS framework was selected as being particularly relevant to this research due to the applicability of the theory to both implementation and KM activities and the prominent focus on context, which we hypothesised would be important in this study of primary care. Integrating four key constructs, the framework specifies that successful implementation is the achievement of implementation goals, resulting from the *facilitation* of an *innovation* with the *recipients* in their (local, organisational and health system) *context* [32]. Having identified limited applications of i-PARIHS in primary care contexts [33, 34], we hoped to make a theoretical contribution to the use of i-PARIHS in the primary care setting.

### Design

This study used two methods: first, secondary analysis of focus groups undertaken in 2013 with three of the four control practices from the MOSAICS trial, and second, interviews of stakeholders within the JIGSAW implementation project, undertaken in 2018, to explore the experience and process of KM.

Focus groups enabled interaction between professional groups within each practice to be captured [35]. The aim of the focus groups was originally to explore the response to the control practice training and explore if this approach had potential for changing practice. The primary ethical approval, methods and analysis for the focus groups are reported elsewhere [27]. Focus groups were conducted by ZP (Consultant Rheumatologist and qualitative researcher), digitally recorded and transcribed verbatim. For this study, secondary analysis of transcripts was undertaken with a focus on KM and perceptions towards early adoption activities.

The themes identified from the secondary analysis of focus group data, alongside existing literature [9] and discussions with a stakeholder workshop (including public contributors), informed the development of topic guides for the interview study (Additional File 1). Topic guides were iteratively modified during the interviews as new findings emerged. Individuals working within, or associated with, general practices involved in JIGSAW (including GPs, academics, PNs, commissioners, patients) were eligible to participate. With written consent, interviews were conducted face-to-face or over the telephone, digitally recorded and transcribed verbatim. Data were collected by LS (physiotherapist and qualitative researcher) from February to September 2018. A snowball sampling technique [36] was initially used, supplemented with a purposive approach to recruit participants who had experienced JIGSAW in at least three different practices and a range of experience (ensuring lay representatives and a variety of professional backgrounds were included), until theoretical saturation was achieved [37].

### Data analysis

Analysis first took an inductive approach that was guided by underpinning literature and theory. Using NVivo 11 [38], after a period of familiarisation, open (inductive) coding took place to generate initial codes. Independent double coding (LS and ZP) of a sample of transcripts was completed. Coding was compared and links with implementation theories discussed. Subsequently, the coding was revised, and two further iterative cycles of constant comparison were undertaken to refine overarching themes and subthemes. This drew on recognised techniques including the scrutiny of deviant cases, checking for confirmatory or challenging evidence within the dataset, and interpreting patterns [39]. Specific analysis meetings took place with authors (LS, ZP, AF (Academic Senior Lecturer of Nursing), KD (Principle Investigator MOSAICS, Chief Investigator JIGSAW)) after each cycle of revisions to reflect upon and discuss the themes and coding framework and to carefully consider any connections between the empirical data and theoretical assumptions. Theoretical hypotheses relating to the data were scrutinised in two further analysis meetings, one with GC (Professor of Public Management) and one with public contributors. A final coding framework was agreed and re-evaluated to ensure the analysis was a true representation of the data; analysis then moved into the next phase as each subtheme was mapped to i-PARIHS constructs. The findings were then critically compared with previous studies that have either contributed to the formulation and development of i-PARIHS or been informed by i-PARIHS [33, 40, 41] to identify any differences or omissions which may suggest new theoretical insights.

### Public contributor involvement

Public contributor involvement is reported according to the GRIPP2 checklist [42]. The Lay INvolvement in Knowledge mobilisation (LINK) Group at Keele University supports meaningful Patient and Public Involvement and Engagement (PPIE) in the implementation of research evidence. The LINK group comprises individuals with experience from a Research User Group (RUG) [43], the Applied Research Collaborative West Midlands, local PPIE groups, ethical review panels, charities (e.g. Versus Arthritis) and healthcare staff and carers. LINK members participated in a stakeholder workshop discussion to inform the interview topic guide development and an analysis meeting to aid interpretation of interview findings.

## Results

Twenty-one multi-disciplinary professionals (fourteen GPs, six PNs, one healthcare support worker) from three of the four MOSAICS trial control practices participated in one of three focus groups (5–8 participants per group), lasting 60–90 min. In the fourth MOSAICS control practice, a mutually agreeable time for practice staff to participate could not be arranged. Thirteen stakeholders participated in semi-structured interviews: five GPs (two with commissioning experience, one clinical-academic), two PNs, a clinical academic physiotherapist, a commissioner, two individuals with project management and managerial roles and two lay individuals (member of LINK group and knowledge broker). Participants collectively had experience of JIGSAW implementation in 60 practices in three counties across the West Midlands, UK. Four men and nine women were interviewed (duration 25 to 110 min). Four individuals did not respond to the study invitation (two clinical and two non-clinical).

Four overarching themes exploring the uptake of the innovations from the MOSAICS research study into the JIGSAW implementation project were identified from analysis of both datasets: the innovation as a motivator for planning implementation, moving from knowing to doing, the influence of the primary care context on KM and the key determinants of optimal KM.

The first two themes were predominantly identified from the focus group data and the latter two from the interview data. Focus group data related to the planning stages of implementation and (in i-PARIHS terms) involved the recipients engaging with the MOSAICS innovation which addressed contextual needs and drivers. Whereas, the interview data related to the operationalisation or ‘doing’ phase of implementation and concerned how the innovation was facilitated into practice and by whom (recipients and facilitators) relevant to local contextual circumstances. As such, the focus group and interview data together give a view of the implementation process across time. Despite the differing study aims and topic guide emphasis, some overlap was shown relating to ‘the innovation as a motivator to implementation’ and ‘moving from knowing to doing’ themes, particularly in the discussion of the innovation and context. A description of each theme and sub-theme, relationship with i-PARIHS, and supporting quotes (identified by Q‘*n*’ in the text) are presented in Table 3 and discussed below.

**Table 3:** Theme descriptions and illustrative quotes

Main theme	Subtheme	Relevant i-PARIHS domains	Description	Illustrative quote(s) and data source
The innovation as a motivator to implementation planning	The nature of the innovation	Innovation Recipients Context Facilitation	Participants acknowledged how the content and delivery of training, research evidence presented during training, and evidence-based explanations facilitated a shift towards addressing an unmet need and how by engaging with training activities such as simulated patients, were helpful. It was not only the formal research evidence that had been packaged and presented in the training, but evidence about patient experience, cost and tacit knowledge held by clinicians and managers that unlocked the potential for implementation to occur. The alignment of the innovation with current policy enabled a way of managing people with OA that supported self-management and moved away from the medical model. The whole practice approach to training was described as ‘unique’ with participants reflecting on the usual lack of time and opportunity within general practice to attend training sessions with their colleagues. This reflected the social norms amongst each practice group and highlighted how rarely primary care practitioners meet to discuss evidence-based practice or implementation of best evidence. For the focus group participants, having training staged over three weeks provided an opportunity to practice staff for reflection and feedback (individually and as a team). This facilitated both changes to individual practice and discussion as to how to implement as a practice team. In addition, it enabled nurses to try out elements of the training in practice and identify how elements of the training were transferrable to other elements of care for long-term conditions, i.e. diabetes.	**Focus groups** **Q1.** It is a very different approach, isn’t it, to the, ‘You’ve got a sore knee - ask for an orthopaedic opinion,’ which is the surgical model. And the training has been very much a primary care management model, which is much more appropriate, and I think that’s been very helpful [P1GP2] **Q2.** It need not be the GP that then takes that forward, I suppose, with trained nurses or train somebody else…We’ve got a new secretary coming…one of the two secretaries is a lady who has done … some sort of fitness programme or something like that… So, there are quite a lot of people are interested [P3GP1] **Q3.** The whole project has been great. It has brought us together on a number of occasions. But often one of us will learn something and then, keep it to yourself and you don’t actually get to, to talk to your partners about it. So, as you all do it at the same time, it’s kind of, unique really, isn’t it? We don’t do that very often…it’s been great you guys coming to talk to us [P3GP1]
	Addressing alternative priorities and drivers	Innovation Recipients Context Facilitation	By attending the training component of the innovation, practices were able to identify a previously unmet need for the care and management for people with OA and how this could be improved. Flexibility was a key feature of the innovation that enabled it to be delivered in more than one way and to fit with local contextual factors and existing organisational systems. A range of contextual factors specific to each practice played a part in influencing implementation. Participants described several examples of individual and practice priorities that influenced implementation and subsequent change. For example, one practice was identified as a financial outlier in the region due to ‘high referrals rates in orthopaedics’. Furthermore, the need and desire to reduce referrals to x-ray and secondary care, meet targets such as Care Quality Commission (CQC), reduce consultations (with orthopaedic surgeons), a positive financial impact, and ability to manage patients with other long-term conditions were cited benefits of the JIGSAW approach. The characteristics and needs of a practices local population influenced engagement with implementation in some practices. Factors such as an elderly, rural population were motivators to implement the JIGSAW approach whereby patient physical mobility was viewed as important. This, in turn, influenced how some individuals perceived and prioritised the knowledge from the NICE guidance.	**Focus groups** **Q4.** The stuff from Keele, gave us permission and for me it validated - I found that the research, the graphs they put up were very useful…to me, it seemed to be that there’s a different potential and a different narrative now. And, that's endorsed by the research we’ve been given. It just seems a better approach all round…what I hadn’t got was the knowledge that what I was saying was actually evidenced based…that was a big endorsement. I found it very helpful [P1GP2] **Q5.** It’s going to reduce – I think it’ll reduce consultations (to secondary care)…it does reduce your other requirements [P3PN2] **Q6.** They showed us how to get around these blocking signals that the patients send out, and that’s been really useful because I’ve used it in other respects [diabetes management] as well [P1PN2] **Interviews** **Q7.** One was, ‘Do you know? It will prompt you to do best care in line with NICE’ and two, ‘When you’ve got a CQC visit coming in...’ – which they were about to have, so the CQC had just announced they were about to start inspecting general practices. They’d never been inspected before, so there were other drivers that give you a bit of a gift… ‘When the CQC come in and say, “How do you know you do Best Care?” For OA, you’ll be able to say, “This template complies with NICE guidance and we can run a report”. ‘You know, it’s up to you’. So, we had a double whammy [P05M]
	Maintaining the ‘balance’ within general practice	Context Innovation	A key consideration for whether a practice would implement the innovation related to the likelihood of the innovation creating more work within the practice at the expense of other conditions and hence disrupting the balance within the practice. This highlighted the pressures faced in general practice and how equipoise is an important consideration in each practice.	**Focus groups** **Q8.** It’s difficult to put it in proportion, I think. You can always improve people’s care, but you can't do it endlessly because you've got, you’ve got other things too, er, it’s general practice, not target practice [P2GP1] **Q9.** The only issue I had with, with all of that, outside of it is the proportionality of it all. You know, obviously, you’re focused on this. I mean, you’re a rheumatologist and you’re focused on osteoarthritis, as well. We’re not focused and, shouldn’t be focused. And it’s one of the issues I have with the whole, the way medicine’s going at the moment, in general, but you have to look at keeping everything balanced, because we’re only human and we can only do a certain amount, and when you’ve got to keep 150,000 balls in the air [P2GP1] **Interviews** **Q10.** If it’s in primary care you’ve got to either fund it or create the funded time for them. If you’re putting something in you’ve got to take something out because they just don’t have the capacity [P11M]
Moving from ‘knowing’ to ‘doing’	N/A	Context Facilitation Recipients	The facilitated focus group discussion (conducted as the end of MOSAICS) was found to be a vehicle for KM in which practices ‘action planned’ implementation in the planning stages. The discussion facilitated implementation next steps and helped practices consider ways in which elements of the training could be incorporated and implemented in each practice. A sense of ownership was described by participants. Characteristics of the practice team, including their attitudes to change and believing in the innovation, were important in optimising implementation. Individual attitudes and characteristics (enthusiasm, motivation) also contributed to driving change. Implementation planning took place collaboratively within the focus groups, however enthusiastic staff members were central to action planning change. One practice suggested that ongoing discussions regarding implementation may not have occurred in the absence of collaboration. This prompted the Impact Accelerator Unit (IAU) to consider appropriate ‘champions’ to engage with and work with practices outside of the context of the research trial and facilitate implementation. Champions with clinical, academic, managerial, leadership expertise were recognised as central to implementation. Clinical champions who had played a part in facilitating implementation described ways to approach implementation in a new general practice and identified the importance of understanding the local context factors	**Focus groups** **Q11.** It’s actually really helpful having an external person in, to kind of, guide us through it and make us think about it in perhaps a different way. So, I think we’ve done it better than we would have done [without the facilitated focus group discussion]…definitely (P3GP1) **Q12.** I can see it fitting in place with a little bit of education, a little bit of exercise from me in that consultation saying like, ‘In two to three weeks’ time I want you to come and see (practice nurse names) to have a bit of follow up just to make sure you’re doing the exercises correctly and err and they’ll just go through a few other things that you can be maybe doing as a, as the next step.’ That could work quite well really [P1GP1] **Q13.** (practice nurse name) and I can have a look at the big pull-out sheet and see if we can section the exercises up and put them on docman…we communicate with the doctors using patient-connected tasks. I mean we could let you know; we could inform the doctors that way, feedback that way if they wanted us to [P1PN1] **Interviews** **Q14.** You need to know a bit about the practice. So, if you sent me out now into (area) to do JIGSAW in a practice I’d never been – well, I don’t know any of the practices. I would make some definite attempt to find out who worked there, what type of special services they offered, what that – their part of (area) was like, what types of patients were they likely to see before I went in. And who – how many nurses they had, so do a bit of homework [P12GP]
The influence of the primary care context on KM	Non-modifiable factors – restricted resource and capacity	Context	External contextual factors included restricted resource and capacity. Participants discussed the primary care context and how this had changed over time., affecting practice income and their confidence to invest in new staff, services, and resources. The political and financial climate was shown to elicit a reluctance to ‘spend money’ as financial savings was often a high priority for practices. Capacity for implementation were suggested to be compounded by a recruitment crisis in primary care, a reduced desire to work in general practice among GPs and high staff turnover which made ongoing training (of new staff) a challenge.	**Interviews** **Q15.** They just keep asking more of us and we haven’t got the time to do that within the team we’ve got [P05M] **Q16.** The climate is changing rapidly. People are more and more reluctant to put their hands in their own pockets to, to fund a service that’s not attracting any funding [P03GP]
	Non-modifiable factors – policy and regulatory environment	Context	Policy and the regulatory environment could affect KM both positively and negatively. Participants described how the increased pressure and demands from policy and regulatory factors (including Care Quality Commission (CQC), Quality and Outcomes Framework (QOF)) have resulted in a ‘target and payment driven’ workforce, and a ‘tick box mentality’ that ‘stifles innovation’. For example, the introduction of the QOF was perceived to influence practice staff views of what a clinical priority was and accentuated the target driven mindset of general practices by driving behaviour and processes to gain financial reward. However, one practice identified JIGSAW in their CQC inspection and described it as a way of showing how their practice was ‘doing something over and above what others are’ for the quality of musculoskeletal care.	**Interviews** **Q17.** I think for a lot of them they sort of say, well it’s a time factor, you know it’s not top of the priority because it doesn’t qualify for QOF and therefore because it’s not on their plan of target hit list it’s very much down the pecking order [P02C] **Q18.** One was, ‘Do you know? It will prompt you to do best care in line with NICE’ and two, ‘When you’ve got a CQC visit coming in...’ – which they were about to have, so the CQC had just announced they were about to start inspecting general practices. They’d never been inspected before, so there were other drivers that give you a bit of a gift… ‘When the CQC come in and say, “How do you know you do Best Care?” For OA, you’ll be able to say, “This template complies with NICE guidance and we can run a report”. ‘You know, it’s up to you’. So, we had a double whammy [P05M]
	Non-modifiable factors –service and system design	Context Recipients Facilitation	The system design was reported to stymie KM by encouraging working in silos and making cross-boundary working challenging. Working in silos was suggested to limit interactions between key stakeholders and resist information sharing. Practices who worked in isolation were suggested to encourage an inward facing approach. Staff who had dual roles were seen to be helpful in facilitating implementation.	**Interviews** **Q19.** Service design is often just a patchwork of, erm, you know, sort of sticking plasters and, and small changes without anybody stepping back and looking at services holistically…I’m seeing loads (of system design barriers) at the moment, in terms of information sharing across organisations, systems, and processes that support clinicians to work in different environments (P07GP)
	Modifiable factors – staffing model	Context Recipients	The variation of staffing models and structure between practices was identified as having the potential to be both a barrier and an enabler to implementation. A trend for fewer partners in practices and more salaried doctors was described, with several participants suggesting that there was a greater chance of successful implementation in practices that adopted a ‘traditional’ partnership model due to staff feeling a sense of ownership.	**Interviews** **Q20.** I’m most familiar with the partnership model, erm, because it’s historical and I guess I feel most comfortable with that because you’ve got a bunch of people who are equals and are colleagues and although you might find it difficult to convince them, once you’ve got the body of people together, you know that they are all going to carry on thinking in the same way and that their management decisions, once they are joint, will be executed. I think you always get refuseniks in a practice so you might think you’ve got everyone on board but actually, there are one or two that don’t want to do it but I think that’s quite an easy model [P08M] **Q21.** If you’re ultimately responsible for your own destiny and your own pay, and your staff, and the welfare of your patients in a small population, I think you’re going to be much more involved in designing that [P03GP]
	Modifiable factors – practice culture	Context Recipients	Participants described how implementation is influenced by several elements of the culture within a general practice such as hierarchy, attitudes towards change, relationships with external partners, communication, leadership and knowledge ‘blockers’. The role of PPG groups in supporting decision making in one general practice was also discussed by several participants. The presence of hierarchy within a practice was reported to impact the social behaviour and cohesiveness of the group working within it. Variability of power and control for different professional groups was described that impacted on knowledge use and mobilisation in practice.	**Interviews** **Q22.** The nurses in the practice are not allowed any free thinking really, they’re very controlled and they have to do what the practice manager says. Whereas in the other practice, they’re more like nurse practitioners [P12GP] **Q23.** Practice nurses have been ignored as a group. They get paid different amounts at different practices, they’re not agenda for change, they’ve no right to CPD, they are employees of a GP practice, so the variation in practice nurse engagement could be huge. We have some practice nurses who didn’t engage at all through to others who absolutely drove it and loved it like it was vocational for them. And you’ve got no leverage over that because the system has left them in a terrible place [P11M]
	Modifiable factors – the role of the patient	Recipients Facilitation Context	The ability of patients to drive change in primary care was suggested to be due to their knowledge and expertise in their condition along with their preferences for how care should be delivered. This was important to clinical and non-clinical participants who described the ways in which patient groups from academic institutions, patient participant groups in practices and in the community could and did influence implementation.	**Interviews** **Q24.** I think that patient groups are perhaps one of the most powerful resources, in terms of pushing change. I don’t see it as coming from above and I’m, I’m reluctant to say it, I don’t think I’d see it coming from the medical profession as much as it has done in the past or might have done. So, I think it needs to come from somewhere and really, the people with the most vested interests are the patients, - for understandable reasons and I think they’ll drive the agenda more than anybody else [P03GP] **Q25.** They’ve played a huge role I would say probably an underutilised one as well again through time, so by connecting with the patient groups, they have become spokespeople so they’re part of the culture change for me. They have been able to articulate that to other patients; you know the change in approach and the reinforcement of understanding about conservative management. And that’s only the start of the journey, you know it needs to go on, but I think they’ve been, for me I felt they were powerful [P11M]
Key determinants of optimal KM	Perceptions and experiences of individuals as mobilisers of knowledge	Facilitation	The value and impact that those who mobilised knowledge had in facilitating implementation of JIGSAW, including the activities undertaken, their skills and attributes both individually and as teams. Mobilisers of knowledge were reportedly essential for optimising the implementation of JIGSAW; clinicians alone were perceived to lack the capacity in some general practices to drive change for OA considering it was often perceived as a low priority. It was reported that KM may be accelerated by the inclusion of an additional facilitator in primary care.	**Interviews** **Q26.** Having a knowledge mobilisation, someone who can broker that information, can make it concise can separate the wheat from the chaff and can get the salient points across in an easy digestible way is important because as a busy clinician you just simply can’t keep up to date… I think having people whose job is dedicated to supporting and facilitating that knowledge mobilisation that might help the process [P01C] **Q27.** I think this is basically about the implementation, is helping people out to transfer from one point, from one stance to the other. And on the way, showing them little gains, just to keep the interest, I guess it’s almost like the salesperson techniques [P13GP] **Q28.** I mean the idea of bringing about more change in a practice that’s struggling to make ends meet and trying to fulfil its obligations to its patients, then I think the idea of more change just doesn’t appeal anymore. I think people are exhausted by too many changes and although this, as I say, is a nice project – really neat, small, not a huge workload –but anything extra, even if it’s – you know, licking stamps to put on envelopes, they’d say no [P03GP] **Q29.** Professionals will take it as their, it’s their job, it’s part of their job to mobilise knowledge between colleagues, to make sure that you know the fellow GPs in their practice know about this new research so it’s natural to them, but patients aren’t given the knowledge in the first place to be able to do it [P04L] **Q30.** I guess it’s giving people, making everybody a patient champion making everybody a person champion, a champion of knowledge, just giving people that information and the encouragement to just go out and talk to others and use their own networks to spread the message wider [P04L]
	Knowledge networks	Facilitation Recipients Context	The ways in which the affiliation to various networks or groups facilitated the transfer of knowledge across organisational, professional and societal boundaries. Including, confidence; problem-solving to overcome barriers; and, a catalyst to decision making.	**Interviews** **Q31.** Very important and, as I said, that created the groundswell of interest simultaneously with what was happening with the clinicians and if anything, possibly more important, because a lot of people were either brothers, friends, of the initial people I spoke to in that PPG group, you know, might be a sister , a mother, a whoever, they kind of then told them about the service, they went in, spoke to their GP, said I’m really interested in hearing more about this or can you refer me to the new physio service [P01C] **Q32.** To have the right people around the table from the beginning from when you’re trying to describe what it is that you want to do because that’s when you’ll pick up what the win, wins are and what the barriers will be [P11M] **Q33.** They really don’t want to know what the research is. We find that a lot. What they want to know is what the cost savings is; how it’s going to affect them and their referral rates and how easy is it to implement. So, I think if there was a business case that speaks that language to commissioners that gives them, ‘this is what it can do for your CCG if you implement it. After 12 months, you’ll be here’ – that kind of thing [P09M] **Q34.** They (networks) provide you with an opportunity to challenge the way that you have been doing things or your perception of the way that you’re doing things. So whether it’s the orthopaedic surgeons, whether it’s somebody from a different area of the country you know, it’s that exposure to people who are asking you why and also listening to how, you know how people have got to where they’ve got, with their progress and implementation. And then in addition to that, it’s that exposure to yeah okay the evidence is there and case studies are there but actually it’s the human narrative. So, the networks for me is about human contact with other people, it gets far more synapses I think than reading something [P11M]
	The workload of KM	Facilitation Context Recipients	The workload associated with KM required for successful implementation (which often was too great for clinicians alone to undertake) and the approaches and people required to facilitate this.	**Interviews** **Q35.** Quite often what they want, when there is a necessity for change, they want you to give them a plan every step of the way. And if I reflect back to how successful MOSAICS was, they were supported to make the change every step of the way and everything was funded but you know right down to the setting up the clinics, the training, when the nurse was out, backfilling the nurse, you guys supported them every step of the way. And once you’ve stepped away actually even when we continue to fund the enhanced service, practices from the first fell off of the participation [P11M]

### The innovation as a motivator for planning implementation

Participants described how the delivery and intended clinical outcomes of the innovation, designed to improve the management of OA, met the needs of their elderly rural population (to whom maintaining mobility was crucial) and addressed practice priorities, such as reducing orthopaedic referral rates. The focus on self-management aligned with health policy and gave them a different option to their existing ‘surgical model’ of referring patients to orthopaedics [Q1]. The innovation was perceived as flexible [Q2], enabling a ‘fit’ between the innovation with the practices’ current service design.

With respect to training, participants valued the whole practice approach [Q3], opportunities for in-practice reflection in between training sessions, and the integration of research evidence. The training validated the approach of giving patients a more positive message about outlook and enabled clinicians to give a detailed, evidence-based explanation of the prognosis of OA [Q4].

Several individual and organisational motivators were described (e.g. enhanced transferrable skills for managing other patient groups) which influenced how some individuals perceived and prioritised the innovation to address NICE guidance for OA [Q5–7]. The training facilitated a shift in perspectives about OA, from it being a condition with a negative outlook, and increased awareness of current suboptimal OA care. Furthermore, the training was delivered by staff from the IAU, some of whom had generated some of the research evidence presented. The reputation of the unit increased trust and credibility.

However, focussing care particularly for one condition or patient group was perceived to have the potential to detrimentally impact on the care of other conditions or groups, suggesting that implementation of an innovation may also disrupt equipoise within a practice [Q8–10]. This potential barrier was not realised as participants identified how managing OA could enhance management of other long-term conditions (LTCs), e.g. by having advice to offer people with diabetes who suggested that arthritis would stop them exercising to lose weight.

### Moving from ‘knowing’ to ‘doing’

In the context of the MOSAICS research study, the focus groups themselves facilitated implementation by enabling recipients to consider the application of knowledge from the training relevant within their practice circumstances and to develop strategies to overcome potential barriers [Q11]. In the context of general practice, participants reported rarely meeting as a group and the need for ‘headspace’ to stop and think about implementing new knowledge. This was complemented by engaged and enthusiastic individuals who took ownership of implementation [Q12, 13].

Considering workload pressures, and that OA was often perceived as a low priority, clinicians alone were perceived to lack the capacity to implement JIGSAW. Facilitation of implementation initiation within JIGSAW was undertaken by a team of multi-disciplinary champions, rather than one individual. Many of the team members had boundary spanning roles, and a detailed understanding of the primary care context, high-quality OA care and the MOSAICS study. Participants described how knowledge of a practice was important for successful implementation [Q14].

### The influence of the primary care context on KM

#### External context

##### Restricted resource and capacity

Capacity for implementation was hindered by a recruitment crisis in primary care, a reduced desire to work in general practice among GPs and high staff turnover which challenged ongoing training. General practice was described as a ‘completely saturated service’. Clinical participants described a perception of unlimited demands whereby they ‘just keep being put upon’ [Q15].

Primary care staff were reportedly hesitant to mobilise new knowledge and pay to implement an intervention that provides no financial savings [Q16]. Consequently, implementation of the JIGSAW innovations only appeared to be acceptable if no additional resources were required. Views about funding associated with implementation varied; on one hand, it provided an incentive for engagement, and on the other, it was irrelevant if implementation barriers were capacity or staff recruitment.

##### Policy and the regulatory environment

Participants described how the increased pressure and demands from policy and regulatory factors have resulted in a ‘target and payment driven’ workforce, and a ‘tick box mentality’ that ‘stifles innovation’ and KM. For example, in some practices, the Quality and Outcomes Framework (QOF) was perceived to influence practice staff views of clinical priorities thus possibly negatively impacting the adoption of JIGSAW, as OA does not have an associated QOF indicator [Q17].

Adherence to NICE guidance for the management of OA alone was not a motivator for the implementation of JIGSAW. However, the idea of evidencing quality care to external regulators (Care Quality Commission domains, e.g. effective care) was used by the IAU as an incentive to promote practice buy-in to implementation [Q18].

##### Service and system design

System design was reported to stymie KM by encouraging working in silos and making cross-boundary working challenging by limiting interactions between stakeholders and impeding information sharing. ‘Knowledge blocks’ (barriers or blocking of knowledge flow) were described within and between organisations and professionals for example, between general practice organisations, between academia and clinical practice and between primary and secondary care [Q19]. The role of ‘champions’ (clinical, managerial, lay and academic) from the IAU, comprising boundary spanning individuals who ‘knew the system’ and could shift thoughts and ‘pull a few strings’, were described as essential for overcoming organisational boundaries.

#### Internal organisational context

##### Staffing model

Having staff on temporary contracts, or with less control over practice business (e.g. salaried vs partnered GPs) hindered implementation [Q20–21]. The extent to which staff have a vested interest in practice performance affected HCPs attitudes towards engagement with KM. A sense of ownership and accountability appeared necessary for staff engagement in implementation.

##### Practice culture

Participants who took pride in their practice culture described their team as ‘forward thinking’, ‘early adopters’, with a ‘can-do attitude’. Practices that valued continual professional development reportedly had a willingness to work together and engage with external partners to mobilise knowledge and implement JIGSAW.

In contrast, other participants described instances of practice culture negatively influencing KM. HCPs who were experiencing change fatigue were perceived to be disengaged with implementation due to work pressure and feeling unable to implement new innovations. In some cases, practice hierarchy and power dynamics were reported to impact the social behaviour and cohesiveness of the staff, whereby, one individual could block or facilitate KM. For example, some PNs had the ambition to lead change, yet perceived they lacked autonomy over decision making with the practice manager or GP partners holding discretion [Q22–23].

##### The role of the patient

Participants described how patients are imperative to driving change in primary care, due to their knowledge and expertise in a condition along with their preference for care delivery [Q24]. Patient involvement was described as essential in achieving successful KM and subsequent implementation of JIGSAW in one practice [Q25]. This was achieved by collaborative working between the practice PPG and the LINK group from the IAU.

### Key determinants of optimal KM

In response to a practice-led demand for implementation, the IAU utilised an array of skills and networks to drive KM and facilitate implementation.

#### Perceptions and experiences of individuals as mobilisers of knowledge

Participants reported how an individual who creates, collates or shares knowledge to facilitate implementation was essential for optimising the implementation of JIGSAW [Q26]. Several participants self-identified or were identified by others as key mobilisers of knowledge. Participants lacked clarity about whose role it is to mobilise knowledge. Some viewed it as everybody’s role, others believed a senior person within an organisation was best suited.

It was suggested that to be successful mobilisers of knowledge required the ability to filter best practice evidence, translate to stakeholders in a meaningful way and frame knowledge for different audiences, described as being good ‘sales reps’ [Q27]. Having an intimate knowledge of the delivery system context and the recipients of KM (including their drivers and priorities) was important to navigate barriers and lever change. One participant described how ‘change fatigue’ [Q28] could be overcome by dedicated mobilisers of knowledge understanding current practice and helping clinicians to efficiently transform services by collaboratively addressing organisational issues.

Mobilisers of knowledge were described as individuals who ‘wore many hats’ and undertook several roles. Many of these participants had a role within the IAU and identified as a researcher, clinician or manager, considering KM activities as a tacit and supplementary part of their role. Lay interviewees assumed that clinicians knew and understood KM as part of their role and had a more advanced status in KM than patients [Q29]. However, non-lay interviewees reported patients and the public as pivotal mobilisers of knowledge. One participant suggested that academia and clinical practitioners more generally were ‘missing a trick’ with patients as mobilisers to communicate messages to others after witnessing the impact of patient champions in JIGSAW [Q30].

#### Knowledge networks

Knowledge networks comprised a range of formal and informal, professional and lay groups that facilitated the transfer of knowledge across organisational, professional and societal boundaries. The IAU often formed the central links to these networks due to the cross-boundary roles of individuals who worked there. These included primary care locality boards and federation groups, professional and social networks, PPGs, the LINK group, conversational circles and professional groups.

Professional knowledge networks associated with the IAU gave the recipients’ confidence in the KM champions driving the implementation of JIGSAW. This was due to the international reputation of the unit in OA expertise, academic leadership and credibility of previous projects. Cross-boundary working of key individuals whose roles overlap an interface of knowledge networks were considered core components of successful KM.

Patient and public networks were instrumental in the implementation of JIGSAW in one practice. This was largely facilitated by the role of PPIE within the IAU who developed a relationship with the practice PPG, working collaboratively to operationalise JIGSAW. Participants described the value of local public interest in the provision of the JIGSAW OA service [Q31]; this followed engagement of The University of the Third Age by the IAU. This became an influential KM network for JIGSAW in one area which reportedly generated a ‘groundswell of interest’ whereby patients were asking GPs for access to the JIGSAW innovation.

Knowledge networks were perceived to enable problem-solving and accelerate decision-making by including all stakeholders from the outset, identifying and circumnavigating challenges and effectively sharing lessons learned with a wide audience [Q32]. Sometimes solutions involved learning the professional ‘language’ that people speak and identifying the barriers, drivers and consequences for implementation for other stakeholders and organisations. As such, champions from the IAU were able to contextualise the knowledge underpinning the innovation and tailor their ‘sales pitch’ for promoting implementation based on the needs and agendas of their audience [Q33–34]. Alternatively, individuals drew upon the skills and extended networks of others to overcome barriers. Several examples of individuals or organisations ‘doing favours’ for others in different contexts were described which represented the ability to circumnavigate challenges and override the system, sometimes by deviating from formal rules or procedures, to create a new pathway for achieving a goal. Furthermore, the team approach to implementation facilitated a common ground for engagement and knowledge sharing which enabled decision-making based on the perspectives of key stakeholders.

#### The workload of KM

Collaboration between the IAU and general practices was identified as a central enabler of KM to implement JIGSAW due to the leadership, resource and infrastructure provided to support the process. Participants described the workload associated with KM and the value of having a central team of people with dedicated (paid) time to organise the activities and interactions to optimise implementation. The workload included: securing funding to enable free training for local practices, writing business plans, working with PPGs to design and implement marketing materials, negotiating contracts to support staff to deliver the innovation, providing IT support and liaising with key decision-makers. The importance of this involvement was illustrated by one participant who described an example whereby sustained implementation of JIGSAW ceased when the IAU stepped away [Q35].

A further source of ‘work’ was the need to collect and present relevant outcome data to stakeholders. Findings indicate a discordance between the evaluation data required by commissioners compared to the academic evaluation measures selected as part of MOSAICS. In JIGSAW, commissioners not only required data relating to cost but also required impact data from across the musculoskeletal pathway. Co-production of implementation plans with all key stakeholders was suggested to ensure appropriate evaluation and sustainable implementation.

## Discussion

**Fig. 1:**
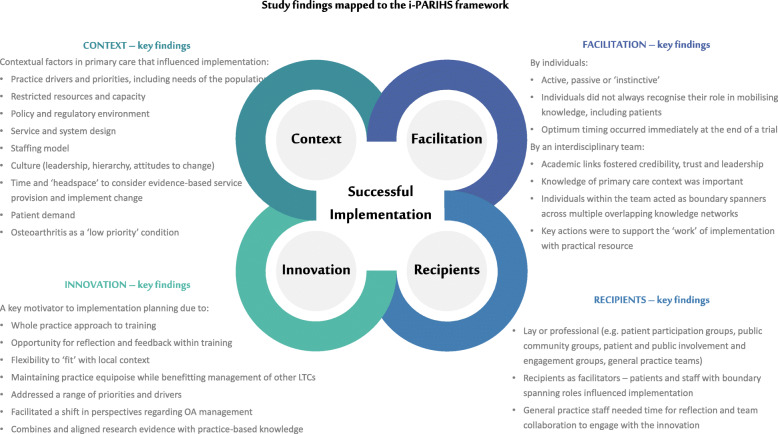
Study findings mapped to the i-PARIHS framework

This study used qualitative methods to investigate the uptake of an innovation for OA in primary care. Findings from secondary analysis of focus group data (collected post-trial with control practices) and interview data (from stakeholders in an implementation project) have identified findings of relevance to all four constructs of the i-PARIHS framework (Fig. 1).

The complex and pressurised *context* of primary care is well recognised [44, 45], making the implementation of new innovations challenging. Whilst the importance of leadership [46–48], impact of hierarchy [48–51] and a positive culture receptive to change [51–53] are commonly cited in primary care literature, our study illustrates the importance of facilitation and innovations in primary care that explicitly address the motivators and priorities of general practices and key stakeholders. This appears to be particularly pertinent when considering OA as a condition which is seen as a ‘low priority’ to patients and clinicians [54, 55] and, therefore, may require more dedicated facilitation from trusted partners. General practice staff often lacked the capacity, skills or autonomy to implement new innovations because the workload burden, balanced with clinical and other practice priorities, was too great. In this study, external facilitation and the innovation itself overcame and addressed these contextual challenges in primary care, by supporting the work of implementation and by providing benefits for other LTCs.

In this study, general practice staff were sometimes reported to lack autonomy to realise desired change. In secondary care contexts, nurses have been reported to lack legitimacy with doctors with regards to sharing knowledge [56]; in this study, PNs had appetite and ambition to drive practice level change but some lacked the autonomy to do so.

Successful implementation was achieved when the *innovation* aligned with a range of stakeholder (recipient) drivers and priorities, only if, practice equipoise was maintained. The innovation was a key motivator for planning implementation as the training, delivered by credible champions (including patients), enabled a shift in perspective of general practice staff regarding the management of OA and realisation that the innovation could improve care. Findings also emphasised how, in primary care, an innovation that incorporated a whole practice approach, including time for reflection, was beneficial for KM. Practice-based learning from the trial highlighted the flexibility of the innovation which enabled the IAU to optimise implementation by addressing a range of priorities to fit local contextual circumstances. For example, PNs engaged with implementation because the training was transferrable to other LTCs.

The i-PARIHS framework identifies recipients as ‘the people who are affected by and influence implementation at both the individual and collective team level’ [32]. In our study, recipients were both lay and professional. We identified how public contributors and lay communities are not only recipients of healthcare innovations but also have potential to be powerful facilitators of implementation, thus illustrating the overlap between the recipient and facilitation constructs of i-PARIHS. Similarly, authors of a process evaluation of the implementation of a mobile phone supported intervention after stroke [57] suggested that the word recipient may emphasise a slightly more passive role for those involved in the implementation process and renamed the recipient construct of i-PARIHS to ‘implementers’.

Public contributors in the LINK group provided insight into how bespoke infrastructure, and processes established within the academic institution were necessary to facilitate their role of supporting PPGs, general practices and implementation researchers to mobilise knowledge for the implementation of JIGSAW. Public contributors in the LINK group often had experience of working in health, regulatory or policy sectors. This suggests that public contributors in implementation may require a different set of skills or experiences to those involved in PPIE for research.

Our findings had most synergy with the *facilitation* construct of i-PARIHS as an ‘active ingredient’ in implementation, illustrating the importance of facilitation in the context of primary care for innovations for OA. Specifically, findings relate to the timing, role and support provided by the facilitator or facilitation. Facilitation varied in type and format and at different times. At the end of the trial, a focus group discussion instigated implementation planning as a mechanism for KM; at this time, the facilitator (an individual) used coaching-style questions to help practices identify the most appropriate, contextually specific ways to operationalise change, the importance of which has been shown in other studies [58]. The opportunity for clinicians to engage in KM via protected time and ‘headspace’, enabling practised-based evidence to guide contextually relevant implementation; the focus group component of the research trial acted as a catalyst for the implementation project. With an increasing demand for designing implementation within research studies, utilising facilitation to support KM at the end of a trial gives a practical and cost-effective approach to embedding implementation planning in trial designs.

Further along the implementation journey, facilitation involved an interdisciplinary team of individuals from the IAU which effectively acted as a knowledge brokering service. That the IAU acted as a knowledge broker reflects that knowledge brokering extends beyond any individual role and organisations can act in a similar fashion. For example, recent large-scale translational investments in Applied Research Collaborations [59] and Allied Health Science Networks [60] in England may similarly enact an organisational level knowledge brokering role [61].

The Unit was internationally renowned for OA research and hence provided academic leadership which facilitated trust and credibility. Individuals from the IAU had an intimate knowledge of the innovation along with the practice setting, context and drivers, were relatable to stakeholders (recipients) and able to ‘sell’ the innovation according to commissioning, clinical and patient needs. Facilitators drew upon practice-based learning from the previous research study (MOSAICS) and their own expertise to enable decision-making based on a combination of formal research-based evidence, local contextual knowledge and tacit knowledge [62]. This has been described as ‘mindlines’ [63] whereby clinicians rely on practice-based experience and base their decisions on internalised and collectively reinforced tacit guidelines (mindlines), informed by interactions with colleagues, opinion leaders and patients. Furthermore, the process whereby formal, research-based knowledge is assimilated with practice-based knowledge, is well described in absorptive capacity theory [64], illustrating how knowledge that is embedded in, and cannot be separated from, practice.

The resources of the IAU provided a solution to primary care staff who wanted to implement JIGSAW but lacked the capacity or skills to do so. The provision of workshops, champions and support, e.g. with business cases, enabled change. Whilst i-PARIHS identifies the skills and focus of facilitators, it does not explicitly address the workload associated with KM and the role of the facilitator in supporting this burden. Normalisation Process Theory (NPT) [29] arguably addresses this concept in more detail by focussing on the ways in which stakeholders work individually and collectively to operationalise an innovation into practice (normalisation) [65, 66].

In our exemplar case, we identified there is not a universal understanding or acknowledgement of the facilitator role. Whilst there is overlap between the roles we identified, the i-PARIHS description of the facilitator role and existing healthcare literature regarding knowledge brokers, we feel subtle differences exist. In contrast to i-PARIHS, where facilitation is described as active or passive, we found a third dimension of ‘instinctive’ facilitation.

Formal or active roles, including researchers in residence and knowledge brokers, who work in a strategic and purposeful way to share knowledge across organisations [67–70], typically involve individuals with facilitatory skills (novice or expert) [33, 40, 71, 72]. Our findings suggest that instinctive facilitation may involve individuals who do not adopt formal brokering roles or fully recognise their role. Successful mobilisers of knowledge in this study included clinical, patient, managerial or academic ‘champions’, with integrated, boundary spanning roles (e.g. clinical academics, a GP partner with a commissioning role) who worked, not individually, but in an interdisciplinary team. Consequently, participants in this study did not have specific facilitator experience. Their understanding of context, including clinical drivers and priorities, was important in enabling facilitation suggesting that the knowledge they bring to bear about mobilising evidence into practice is tacit [73]. Such knowledge might prove difficult to articulate as it is so deeply embedded in collective practice of the clinical community [74]. Public contributors also fulfilled this role, illustrating the potential impact of PPGs, community and lay groups in facilitating implementation. With an increasing range of terminology used to describe the potential role of the facilitator (knowledge broker, implementer, boundary spanner), we suggest that there may be a need for more clear definition and descriptions of these roles to promote consistency and increase visibility. In essence, we highlight that knowledge brokering and facilitation roles, however we label them, are not always formalised, as implementation science literature might suggest [75].

This study adopted the i-PARIHS framework to inform data analysis and careful consideration has been taken to incorporate a theoretically informative approach to explain what the results of the empirical findings mean for the development and further understanding of the theory [31]. Further strengths of the work include the broad range of individuals accessing professional and lay perspectives from academic and clinical settings. The topic guides were developed using stakeholder input, existing literature [9] and theory [76] and developed iteratively; public contributors aided interpretation of the results. A robust approach to data analysis, including double coding enhances the trustworthiness of the findings.

A potential limitation is the focus of the study on empirical data that was grounded in a single research study and subsequent implementation project. As a result, the transferability of these findings may be limited; however, we believe the study has relevance to other implementation activities where the innovation relates to guideline implementation, nurse-led care, LTCs and non-prioritised conditions. Our role may have influenced our interpretations, but we endeavoured to remain reflexive throughout by keeping a detailed audit trail of analytical decisions and discussing findings with the broader study team. The collective views of practices and individuals that were not implementing JIGSAW were underrepresented and as a result, the data presented in this account may not offer full insights into barriers and facilitators. However, several participants spoke of unsuccessful attempts to mobilise knowledge in other practices and described the challenges experienced.

Unfortunately, it was not feasible within our timeframe or ethical permissions to triangulate our findings with other sources of data such as quantitative process measures, observations or documentary analysis. We did not conduct longitudinal interviews which would have enabled a more in-depth study of the experience of JIGSAW participants; however, the inclusion of the focus group data collected at the very start of implementation post-trial did result in a breadth of findings at different time points.

## Conclusion

This study explored KM from a trial to implementation for OA in primary care and has contributed to the development of the i-PARIHS framework by building on previous theoretical knowledge. This study identified (1) the role of an inter-disciplinary knowledge brokering service nested within a clinical-academic unit of expertise to support implementation of OA innovations in primary care by understanding the primary context and providing practical support and resource; (2) how individuals who mobilise knowledge, without explicit KM roles, can facilitate change if they are trusted and credible to recipients; (3) that the end of a trial is a timely opportunity for mobilising knowledge and implementation planning; and (4) that patients and the public can be both recipients and facilitators of implementation; however, support in this role, including a supportive infrastructure, is needed. Further work is needed to define and clarify non-expert facilitation roles, including the role of patient contributors, and explore the transferability of knowledge brokering services within academic units in other contexts.

## Supplementary Information

**Additional file 1:.** Interview Topic Guide

## Data Availability

The School for Primary, Community and Social Care, Keele University, is committed to sharing access to our anonymised research data derived from our population, consultation, clinical and RCT cohorts. Researchers wanting to apply for access to data from archived studies hosted by the School of Primary, Community and Social care should first email primarycare.datasharing@keele.ac.uk

## Abbreviations

GP: General practitioner
HCPs: Healthcare professionals
IAU: Impact Accelerator Unit
i-PARIHS: The Integrated Promoting Action on Research Implementation in Health Services framework
JIGSAW: Joint Implementation of Guidelines for Osteoarthritis in the West Midlands
KM: Knowledge mobilisation
LINK: Lay INvolvement in Knowledge mobilisation
MOSAICS: Managing OSteoArthritis in ConsultationS
NICE: National Institute for Health and Care Excellence
NPT: Normalisation Process Theory
OA: Osteoarthritis
PPG: Patient Participation Group
PPIE: Patient and Public Involvement and Engagement
PNs: Practice nurses
QOF: Quality and Outcomes Framework

## References

[1] Murray CJ, Vos T, Lozano R, Naghavi M, Flaxman AD, Michaud C (2013). Disability-adjusted life years (DALYs) for 291 diseases and injuries in 21 regions, 1990–2010: a systematic analysis for the Global Burden of Disease Study 2010. Lancet..

[2] Sakellariou G, Conaghan PG, Zhang W, Bijlsma JW, Boyesen P, D’agostino MA, et al. EULAR recommendations for the use of imaging in the clinical management of peripheral joint osteoarthritis. Ann Rheum Dis. 2017:annrheumdis-2016-210815. 10.1136/annrheumdis-2016-210815 28389554

[3] McAlindon TE, Bannuru RR, Sullivan M, Arden N, Berenbaum F, Bierma-Zeinstra S (2014). OARSI guidelines for the non-surgical management of knee osteoarthritis. Osteoarthr Cartil.

[4] Porcheret M, Jordan K, Croft P (2007). Treatment of knee pain in older adults in primary care: development of an evidence-based model of care. Rheumatology..

[5] Allen KD, Choong PF, Davis AM, Dowsey MM, Dziedzic KS, Emery C (2016). Osteoarthritis: models for appropriate care across the disease continuum. Best Pract Res Clin Rheumatol.

[6] Dziedzic KS, Healey EL, Porcheret M, Afolabi EK, Lewis M, Morden A (2018). Implementing core NICE guidelines for osteoarthritis in primary care with a model consultation (MOSAICS): a cluster randomised controlled trial. Osteoarthr Cartil.

[7] Bauer MS, Damschroder L, Hagedorn H, Smith J, Kilbourne AM (2015). An introduction to implementation science for the non-specialist. BMC Psychol.

[8] Swaithes L, Paskins Z, Dziedzic K, Finney A. Factors influencing the implementation of evidence-based guidelines for osteoarthritis in primary care: a systematic review and thematic synthesis. Musculoskeletal Care. 2020. 10.1002/msc.1452 31997576

[9] Lau R, Stevenson F, Ong BN, Dziedzic K, Treweek S, Eldridge S (2016). Achieving change in primary care—causes of the evidence to practice gap: systematic reviews of reviews. Implement Sci.

[10] Lau R, Stevenson F, Ong BN, Dziedzic K, Treweek S, Eldridge S (2015). Achieving change in primary care—effectiveness of strategies for improving implementation of complex interventions: systematic review of reviews. BMJ Open.

[11] Lineker SC, Husted JA (2010). Educational interventions for implementation of arthritis clinical practice guidelines in primary care: effects on health professional behavior. J Rheumatol.

[12] Sharp CA, Swaithes L, Ellis B, Dziedzic K, Walsh N. Implementation research: making better use of evidence to improve healthcare. Rheumatology. 2020 (2020; 0):1-3. 10.1093/rheumatology/keaa088 32252071

[13] Davies HT, Powell AE, Nutley SM. Mobilising knowledge to improve UK health care: learning from other countries and other sectors–a multimethod mapping study. Health Serv Delivery Res. 2015;3(27). 26110190

[14] Ferlie E, Crilly T, Jashapara A, Peckham A (2012). Knowledge mobilisation in healthcare: a critical review of health sector and generic management literature. Soc Sci Med.

[15] Gabbay J, le May A, Jefferson H, Webb D, Lovelock R, Powell J (2003). A case study of knowledge management in multiagency consumer-informed communities of practice: implications for evidence-based policy development in health and social services. Health..

[16] Dziedzic KS, Healey EL, Porcheret M, Ong BN, Main CJ, Jordan KP (2014). Implementing the NICE osteoarthritis guidelines: a mixed methods study and cluster randomised trial of a model osteoarthritis consultation in primary care-the Management of OsteoArthritis In Consultations (MOSAICS) study protocol. Implement Sci.

[17] NICE (2014). Osteoarthritis care and management in adults.

[18] Porcheret M, Grime J, Main C, Dziedzic K (2013). Developing a model osteoarthritis consultation: a Delphi consensus exercise. BMC Musculoskelet Disord.

[19] Porcheret M, Main C, Croft P, McKinley R, Hassell A, Dziedzic K (2014). Development of a behaviour change intervention: a case study on the practical application of theory. Implement Sci.

[20] Grol R, Wensing M, Eccles MP (2004). Improving patient care: implementing change in clinical practice.

[21] Michie S, Johnston M, Abraham C, Lawton R, Parker D, Walker A. Making psychological theory useful for implementing evidence based practice: a consensus approach. Qual Saf Health Care. 2005;14. 10.1136/qshc.2004.011155 PMC1743963 15692000

[22] Dziedzic KS, French S, Davis AM, Geelhoed E, Porcheret M (2016). Implementation of musculoskeletal Models of Care in primary care settings: Theory, practice, evaluation and outcomes for musculoskeletal health in high-income economies. Best Pract Res Clin Rheumatol.

[23] Keele University ARU, National Institute for Health Research (2014). A guide for people who have osteoarthritis.

[24] Healey EL, Main CJ, Ryan S, McHugh GA, Porcheret M, Finney AG (2016). A nurse-led clinic for patients consulting with osteoarthritis in general practice: development and impact of training in a cluster randomised controlled trial. BMC Fam Pract.

[25] Healey E, Main C, Ryan S, McHugh G, Finney A, Dziedzic K (2014). A model osteoarthritis consultation within primary care: a novel nurse-led approach to promote self-management. Ann Rheum Dis.

[26] Edwards JJ, Jordan KP, Peat G, Bedson J, Croft PR, Hay EM, et al. Quality of care for OA: the effect of a point-of-care consultation recording template. Rheumatology. 2014:keu411. 10.1093/rheumatology/keu411 PMC4416084 25336538

[27] Hay E, Dziedzic K, Foster N, Peat G, Bartlam B, Blagojevic-Bucknall M (2018). Optimal primary care management of clinical osteoarthritis and joint pain in older people: a mixed-methods programme of systematic reviews, observational and qualitative studies, and randomised controlled trials. Program Grants Appl Res.

[28] Porcheret M, Main C, Croft P, Dziedzic K (2018). Enhancing delivery of osteoarthritis care in the general practice consultation: evaluation of a behaviour change intervention. BMC Fam Pract.

[29] May C, Finch T, Mair F, Ballini L, Dowrick C, Eccles M, et al. Understanding the implementation of complex interventions in health care: the normalization process model. BMC Health Serv Res. 2007;7. 10.1186/1472-6963-7-148 PMC2089069 17880693

[30] Dziedzic K, Healey E, Porcheret M, Afolabi E, Lewis M, Morden A (2017). Implementing core nice guidelines for osteoarthritis in primary care with a model consultation (MOSAICS): A cluster randomised controlled trial. Osteoarthr Cartil.

[31] Kislov R, Pope C, Martin GP, Wilson PM (2019). Harnessing the power of theorising in implementation science. Implement Sci.

[32] Harvey G, Kitson A. PARIHS revisited: from heuristic to integrated framework for the successful implementation of knowledge into practice. Implement Sci 2016;11(1):1–13. 10.1186/s13012-016-0398-2 PMC4807546 27013464

[33] Laycock A, Harvey G, Percival N, Cunningham F, Bailie J, Matthews V (2018). Application of the i-PARIHS framework for enhancing understanding of interactive dissemination to achieve wide-scale improvement in Indigenous primary healthcare. Health Res Policy Syst.

[34] Wray LO, Ritchie MJ, Oslin DW, Beehler GP (2018). Enhancing implementation of measurement-based mental health care in primary care: a mixed-methods randomized effectiveness evaluation of implementation facilitation. BMC Health Serv Res.

[35] Kitzinger J (1994). The methodology of focus groups: the importance of interaction between research participants. Soc Health Illness.

[36] Bryman A (2008). Social Research Methods.

[37] Silverman D (2013). Doing qualitative research: a practical handbook: SAGE Publications Limited.

[38] Tesch R (2013). Qualitative Types: Analysis Typ: Routledge.

[39] Miles MB, Huberman AM (1994). Qualitative data analysis: an expanded sourcebook: sage.

[40] Harvey G, Llewellyn S, Maniatopoulos G, Boyd A, Procter R (2018). Facilitating the implementation of clinical technology in healthcare: what role does a national agency play?. BMC Health Serv Res.

[41] Yakovchenko V, Bolton RE, Drainoni M-L, Gifford AL (2019). Primary care provider perceptions and experiences of implementing hepatitis C virus birth cohort testing: a qualitative formative evaluation. BMC Health Serv Res.

[42] Staniszewska S, Brett J, Simera I, Seers K, Mockford C, Goodlad S (2017). GRIPP2 reporting checklists: tools to improve reporting of patient and public involvement in research. Res Involve Engage.

[43] Jinks C, Carter P, Rhodes C, Beech R, Dziedzic K, Hughes R (2013). Sustaining patient and public involvement in research: a case study of a research centre. J Care Serv Manag.

[44] Baird B, Reeve H, Ross S. Innovative models of general practice: The King’s Fund; 2018.

[45] Baird B, Charles A, Honeyman M, Maguire D, Das P (2016). Understanding pressures in general practice.

[46] Rycroft-Malone J, Burton C, Wilkinson J, Harvey G, McCormack B, Baker R, et al. Collective action for knowledge mobilisation: a realist evaluation of the Collaborations for Leadership in Applied Health Research and Care. Health Serv Delivery Res. 2015;3(44). 26677504

[47] DiCenso A, Bryant-Lukosius D, Martin-Misener R, Donald F, Abelson J, Bourgeault I (2010). Factors enabling advanced practice nursing role integration in Canada. Nurs Leadersh.

[48] Currie G, Spyridonidis D (2019). Sharing leadership for diffusion of innovation in professionalized settings. Hum Relat.

[49] McInnes S, Peters K, Bonney A, Halcomb E (2017). Understanding collaboration in general practice: a qualitative study. Fam Pract.

[50] Sangster-Gormley E, Martin-Misener R, Downe-Wamboldt B, DiCenso A (2011). Factors affecting nurse practitioner role implementation in Canadian practice settings: an integrative review. J Adv Nurs.

[51] Weiner B. A theory of organizational readiness for change. Implement Sci. 2009;4. 10.1186/1748-5908-4-67 PMC2770024 19840381

[52] Leatt P, Shea C, Studer M, Wang V (2006). IT solutions for patient safety—best practices for successful implementation in healthcare. Healthc Q.

[53] Rutherford J, Leigh J, Monk J, Murray C (2005). Creating an organizational infrastructure to develop and support new nursing roles–a framework for debate. J Nurs Manag.

[54] Egerton T, Diamond L, Buchbinder R, Bennell K, Slade S. A systematic review and evidence synthesis of qualitative studies to identify primary care clinicians’ barriers and enablers to the management of osteoarthritis. Osteoarthr Cartil. 2016. 10.1016/j.joca.2016.12.002 27939622

[55] Paskins Z, Sanders T, Croft PR, Hassell AB (2015). The identity crisis of osteoarthritis in general practice: a qualitative study using video-stimulated recall. Ann Fam Med.

[56] Currie G, Burgess N, Hayton JC (2015). HR practices and knowledge brokering by hybrid middle managers in hospital settings: the influence of professional hierarchy. Hum Resour Manag.

[57] Teriö M, Eriksson G, Kamwesiga JT, Guidetti S (2019). What’s in it for me? A process evaluation of the implementation of a mobile phone-supported intervention after stroke in Uganda. BMC Public Health.

[58] Allen KAM, Dittmann KR, Hutter JA, Chuang C, Donald ML, Enns AL, et al. Implementing a shared decision-making and cognitive strategy-based intervention: knowledge user perspectives and recommendations. J Eval Clin Pract. 2019. 10.1111/jep.13329 31828869

[59] Soper B, Hinrichs S, Drabble S, Yaqub O, Marjanovic S, Hanney S (2015). Delivering the aims of the collaborations for leadership in applied health research and care: understanding their strategies and contributions.

[60] AHSN (2019). Academic Heath Science Networks: The AHSN Network.

[61] Currie G, El Enany N, Lockett A (2014). Intra-professional dynamics in translational health research: The perspective of social scientists. Soc Sci Med.

[62] Green LW (2008). Making research relevant: if it is an evidence-based practice, where’s the practice-based evidence?. Fam Pract.

[63] Gabbay J, le May A (2004). Evidence based guidelines or collectively constructed “mindlines?” Ethnographic study of knowledge management in primary care. Br Med J.

[64] Zahra SA, George G (2002). Absorptive capacity: a review, reconceptualization, and extension. Acad Manag Rev.

[65] May C, Mair FS, Finch T, MacFarlane A, Dowrick C, Treweek S, et al. Development of a theory of implementation and integration: normalization Process Theory. Implement Sci. 2009;4. 10.1186/1748-5908-4-29 PMC2693517 19460163

[66] Murray E, Treweek S, Pope C, MacFarlane A, Ballini L, Dowrick C, et al. Normalisation process theory: a framework for developing, evaluating and implementing complex interventions. BMC Med. 2010;8. 10.1186/1741-7015-8-63 PMC2978112 20961442

[67] Marshall M (2014). Researchers-in-Residence: a solution to the challenge of evidence-informed improvement?. Prim Health Care Res Dev.

[68] Kislov R (2012). Multiprofessional communities of practice in a large-scale healthcare knowledge mobilisation initiative: a qualitative case study of boundary, identity and knowledge sharing: The University of Manchester (United Kingdom).

[69] Lomas J. The in-between world of knowledge brokering. Br Med J. 2007;334. 10.1136/bmj.39038.593380.AE PMC1779881 17235094

[70] Currie G, Spyridonidis D, Oborn E. The influence of HR practices upon knowledge brokering in professional organizations for service improvement: addressing professional legitimacy and identity in health care. Hum Resour Manag. 2019.

[71] Pighills A, Tynan A, Furness L, Rawle M (2019). Occupational therapist led environmental assessment and modification to prevent falls: review of current practice in an Australian rural health service. Aust Occup Ther J.

[72] Byrnes A, Young A, Mudge A, Banks M, Clark D, Bauer J (2018). Prospective application of an implementation framework to improve postoperative nutrition care processes: Evaluation of a mixed methods implementation study. Nutr Diet.

[73] Polanyi M. The tacit dimension: University of Chicago press; 2009.

[74] Lave J, Wenger E. Situated learning: Legitimate peripheral participation: Cambridge University press; 1991.

[75] Rowley E, Morriss R, Currie G, Schneider J (2012). Research into practice: collaboration for leadership in applied health research and care (CLAHRC) for Nottinghamshire, Derbyshire, Lincolnshire (NDL). Implement Sci.

[76] Nilsen P (2015). Making sense of implementation theories, models and frameworks. Implement Sci.

