# Supplementary material for: Understanding the uptake of a clinical innovation for osteoarthritis in primary care: a qualitative study of knowledge mobilisation using the i-PARIHS framework
Source: Implement Sci. 2020 Oct 28;15:95. doi: 10.1186/s13012-020-01055-2 (PMC7594414; doi:10.1186/s13012-020-01055-2)
Supplement: Supplementary file 1 — Additional file 1:. Interview Topic Guide [file 13012_2020_1055_MOESM1_ESM.docx]

**Additional File 1 – Interview Topic Guide**

The initial guide contained broad questions focussing on the following, as well as more discipline specific questions.

- The participants' role and involvement with MOSAICS or JIGSAW
- Experiences of implementation from MOSAICS to JIGSAW
- Perceptions and beliefs relating to barriers and facilitators of the process
- Perceptions and beliefs relating to factors affecting KM and implementation in general practice organisations

In light of the literature review, a stakeholder advisory group, analysis of existing focus group data and PPIE, the topics were progressively refined and refocussed. This reflects the emergent process using an abductive research strategy whereby theory emerges from the data at each progressive stage and the topic guide is refined to reflect this.

A bespoke topic guide was then developed for each interview based on emergent findings and considering the professional discipline of the participant. An example of the topic guide for clinical staff is outlined below.

**Example of a topic guide for a clinical participant.**

Can you start by telling me a bit about (this process) your involvement (as a practice/clinician) with JIGSAW?

What motivated you to get involved?

- *Explore previously unmet need*
- *What was it about the intervention?*
- *Were there any aspects that you were more reserved/concerned about? Balance within the practice, staff training, staff capacity, practice priorities, enthusiasm for change*

What features within the practice influenced implementation?

- Consider culture, leadership, key individuals
- Why do you think that was?
- What is it that motivates or drives your practice to implement things like this? £, patient care, referral rates to x-ray/ortho, patient demographic
- What did the training do? Allow headspace, discussion, reduce hierarchy? Group decisions, transformation? Explore whole practice approach to training

Have you encountered any barriers?

What is stopping other practices taking this up?

What’s happening now?

- *Is it still going? Has it changed? Why?*
- *Has it been monitored? Evaluated?*
- *Have you shared learning with others? CQC?*
- *Who/what are facilitators to this?*
- *Who are the key people involved with driving forwards implementation within your practice? why? What is it about leadership within the practice?*

*Can you suggest anyone else who has been influential in the process who would be beneficial for me to talk to about implementation?*

How do you or your practice find out about new ideas or projects, and how do you decide whether to implement them?

- *What forums or mechanisms do you have to communicate ideas for change? (someone else I’ve interviewed mentioned whatsapp)*
- *Whose responsibility do you think it is to flag ideas for change and then drive those forwards?*
- What strategies do you use in practice to make changes/Implement new ideas?
- *Other examples?* *have you made any changes like this for other conditions? What was similar/different?*

What role do patients play in changing practice and implementation?

- *How were patients involved in this example?*
- *How do you think this could be improved locally and nationally?*
